# Study on Improving Loess Properties with Permeable Polymer Materials

**DOI:** 10.3390/polym14142862

**Published:** 2022-07-14

**Authors:** Jiaqi Mu, Jianqi Zhuang, Jiaxu Kong, Shibao Wang, Jie Wang, Jia Zheng, Yuting Fu, Chenhui Du

**Affiliations:** Key Laboratory of Western China Mineral Resources and Geological Engineering, College of Geological Engineering and Geomatics, Chang’an University, Xi’an 710054, China; m578689985@163.com (J.M.); 18829014384@163.com (J.K.); wangshibao1216@163.com (S.W.); king_jie@163.com (J.W.); zjia0103@163.com (J.Z.); fuyuwenwen@163.com (Y.F.); chenhui_du@chd.edu.cn (C.D.)

**Keywords:** loess, polymer stabilizer, mechanical mechanism, water stability, microstructure

## Abstract

Loess has strong water sensitivity, strong collapsibility, and low strength resulting in failures such as landslides, due to its loose structure. In order to improve the loess characteristics and to better meet the needs of engineering, a colorless, transparent, and permeable composite material is proposed in this paper. Water stability, erosion, unconfined compression, and triaxial tests were conducted to investigate the change of the strength properties and soil erosion resistance. The water sensitivity and strength properties of the loess are significantly improved as the stabilizer concentration increases. When scoured for 20 min, the erosion rates of the reinforced and the unreinforced soil were 95% and 6.25%, respectively, and demonstrated a 15.12 times reduction in erosion rates. The optimal concentration of the mixed solution is 0.6%. The triaxial test, CT, and SEM scanning tests were used to reveal the intrinsic mechanisms. The results demonstrated that the internal friction angle of the reinforced soil increases from 28.09° to 30.57°, and the cohesion changes from 25 kPa to 37.4 kPa. A large number of pores with a diameter of 900–1000 μm are reduced to 0–200 μm, and some pores with a length greater than 600 μm reduce to a length of less than 200 μm; The agglomeration and cementation, the filling of pores, and the formation of membrane structures have contributed greatly to the improvement of loess properties. Furthermore, the newly composite material has significant application potential needed to stabilize soil.

## 1. Introduction

Loess is widely distributed worldwide, especially in the middle reaches of the Yellow River in China, forming the large area of the Loess Plateau, with 6.6% of the total area being covered by loess [1]. Loess is characterized by low strength, high water sensitivity, high compressibility, and strong collapsibility, which has an adverse effect on engineering and the economy [2,3,4]. On the one hand, the loose accumulation of loess is more prone to natural disasters, such as slope instability, landslide, collapse, and debris flow while under the influence of natural factors and human activities [5,6]. Moreover, loess is vulnerable to erosion [7], and the formation of a large number of gullies provides a prerequisite for the occurrence of secondary disasters [8,9,10,11]. In addition, due to the loess’s unique structure, the loess area’s geological environment is extremely fragile; thus, engineering problems such as foundation settlement and tunnel collapse are often encountered [12,13,14]. Therefore, it is vital to improve the properties of loess so that it can better meet engineering requirements.

Currently, the soil reinforcement methods include physical reinforcement and chemical reinforcement. Physical reinforcement methods include dehydration [15,16] and densification [17,18,19,20,21]. Due to the large disturbance of soil, high cost, and hazardous environment, physical reinforcement is no longer suitable for sustainable development [22]. Traditional chemical additives, such as Portland cement, lime, silica fume, fly ash, blast furnace slag, steel slag, and cement kiln dust have been implemented to improve soil [23,24,25,26,27,28,29]. The extensive use of such materials brings severe environmental problems, such as large consumption of nonrenewable resources and high carbon dioxide emissions.

Due to the growing concerns concerning environmental protection, scholars have embarked on numerous explorations in order to seek new environmentally friendly materials. Moreover, significant progress has already been made [30,31,32,33]. In recent years, polymers have attracted significant attention as an alternative material for soil stabilization due to their sustainable advantages. For example, Yang et al. [34] used a modified polypropylene fiber and cement as reinforcement materials to explore the mechanical properties of reinforced loess and to analyze the failure characteristics of the soil. Xanthan gum, polyacrylamide, and resins are new chemical soil stabilizers. These stabilizers contain large numbers of macromolecular chains and hydrophilic groups; the membrane structure formed through the interaction with the soil effectively improves its strength properties as well as the stability behaviors [35,36,37,38]. After mixing a polyurethane polymer with sand, the failure mode of the specimen gradually changes from brittleness to ductile, and the tensile and compressive strength increases linearly with an increase in polymer content [39,40]. Modified sodium carboxymethyl cellulose has been widely used in the study of sand and gravel soil. Furthermore, after reinforcement, the hydraulic properties of the soil greatly improved [41,42]. When polyol prepolymer and fibers were added to the soil as a new material, the research demonstrated that the material significantly improved the soil’s freeze-thaw and evaporation resistance and promoted the plants’ growth [43,44]. Continuous in-depth research of new and novel materials as well as polymer technological advancements play an important role in soil reinforcement. However, there are few studies on applying composite materials to strengthen loess. The majority of the stabilizers are too viscous to automatically penetrate the soil, and can only be mixed with soil by manually stirring during laboratory tests; therefore, it is difficult to popularize and use these materials in a real-world application.

To address the shortcoming of the current research, this study aims to propose a colorless, clear, high-permeability composite stabilizer and evaluate the feasibility of its loess improvement abilities. Water stability, unconfined compression, triaxial and erosion tests were conducted to study the water stability and mechanical properties of loess before and after reinforcement with different polymer concentrations. The characterizations performed by XRD diffraction, CT, and SEM scanning tests were used to reveal the intrinsic mechanisms. The research results provide a reference value for alleviating soil and water loss, engineering construction, disaster prevention, and control within the loess area, therefore providing a theoretical basis for the practical application of the new stabilizer.

## 2. Materials and Methods

### 2.1. Materials

**Figure 1:**
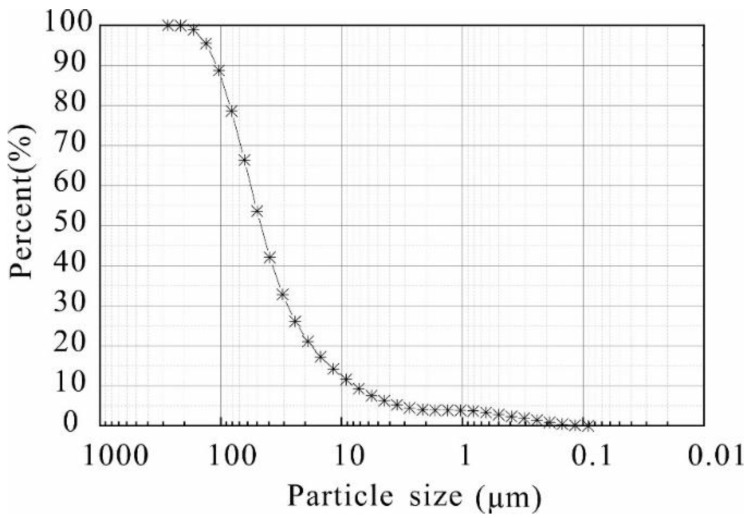
The grain size grading curve of the loess.

The used loess is derived from the backfill area of the Huoxianggou, Xifeng District, Qingyang City, Gansu Province. Shallow loess without surface plant roots is selected for testing. The soil depth in this study is 2 m. The basic physical indexes of the soil are measured according to geotechnical test standards, and the results are presented in Table 1. The soil particle size distribution curve was measured using a bettersize 2000 laser particle size distribution instrument and the results are displayed in Figure 1, in which the contents of sand (>0.02 mm), silt (0.02 mm–0.002 mm), and clay (<0.002 mm) are defined as 36.67%, 50.27%, and 13.06%, respectively.

The polymer stabilizer is synthesized using hydroxypropyl methyl cellulose (A), Starch sodium octenyl succinate (B) and trisodium citrate dihydrate (C), where A and B are organic polymer materials, and C is an inorganic compound. Xi’an Xinhui Building Materials Technology Co., Ltd., Shaanxi, China, supplied all the chemicals used in this study. Physical properties are shown in Table 2.

### 2.2. Specimen Preparation

Test specimens were prepared using the following process. First, the tested soil was air-dried at 100 °C, crushed, and passed through a 2 mm sieve. Second, a specific amount of distilled water was added to the dry soil in order to keep the moisture content at 5%; subsequently, the soil with a moisture content of 5% was placed into the mold to obtain a tested sample with a dry density of 1.5 g/cm^3^ via static pressure. Finally, the soil stabilizer with different concentrations (0%, 0.2%,0.4%, 0.6%, and 0.8%) were sprayed on the surface of the sample and sealed for 24 h to ensure that the stabilizer evenly penetrated into the sample.

### 2.3. Test Progress

#### 2.3.1. Water Stability Test

The water stability test was used to evaluate the process and extent of the influence of water on the sample. Water is an important inducing factor of geological disasters in the loess region; therefore, the study of reinforced soil disintegration has vital practical significance. Samples of different concentrations were immersed in a beaker containing the same amount of water, thus ensuring that the samples were completely submerged in water. Next, we record the failure form and disintegration degree of the sample at different points. The sample diameter is 61.9 mm and the height is 40 mm. In this study, the calculation formula of disintegration rate is as follows:

(1) $$A_{t}=\frac{M-M_{t}}{M}\times 100\%$$

where $A_{t}$(%) = the disintegration rate of the specimen at the time *t*, M(g) = the initial mass of the specimen,$\;M_{t}$ = the residual mass of the specimen at the time *t*.

#### 2.3.2. Unconfined Compression Test and Permeability Test

Unconfined compression and permeability tests were carried out in accordance with the standard for geotechnical test methods [45]. The UCS tests were performed using a YYW-2 strain control type unconfined pressure gauge, it controls the rate of the plate of the unconfined pressure gauge at a controlled rate of 2.4 mm/min. The axial stress of samples was recorded, and the measurement process ended until the specimen is completely destroyed. The average value of three test results is used as a data point. The sample specifications for the tests are 39.1 mm in diameter and 80 mm in height. In the permeability test, the soil was prepared as a cylindrical shaped specimen, the sample specifications are 61.8 mm in diameter and 40 mm in height. The permeability coefficient was calculated for each specimen for six different starting water heads, and the average value was used for data analysis. The unconfined compression and permeability testing device was manufactured by Xi’an Zonghui Instrument Factory Co., Ltd. (Xi’an, China).

#### 2.3.3. Triaxial Test

The TFB-1 unsaturated soil stress–strain controlled triaxial apparatus obtained by Nanjing Zhongzhiyan measurement and Control Technology Co., Ltd. (Nanjing, China) is used for the test. The TFB-1 device can perform triaxial tests on saturated and unsaturated soils under stress–strain conditions. The parameter settings and data acquisition are controlled via computer. The sample specifications are 39.1 mm in diameter and 80 mm in height. The tests were performed with a constant loading rate of 2.4 mm/min under unconsolidated and undrained conditions, and the sampling interval was 0.1 mm.

#### 2.3.4. Erosion Test

The independently designed launder was employed for the erosion tests, which can control both the slope and the flow rate. In order to better simulate a real-world slope, we constructed a chute slope at 30°, with a flow path set at 1 m and a water flow rate of 20 mL/s. During the test preparation stage, the soil with a moisture content of 5% was loaded into the groove. The soil is divided into two parts with equal volumes via a diaphragm. Next, distilled water and 0.6% reinforcing agent are sprayed on the left and right soil surfaces for 24 h to ensure that the stabilizer penetrates evenly into the soil. The eroded soil was collected, oven-dried, and weighted during the process; thus, the erosion loss can be recorded at different times. The erosion ratio was introduced to evaluate the erosion resistance capacity of the soil before and after reinforcement and is defined as:

(2) $$H=\frac{m_{t}}{m_{0}}\times 100\%$$

where *H* (%) = the erosion ratio, *m_t_* (g) = the dry weight of the eroded soil in the *t* minute, and *m*_0_ (g) = the dry weight of all the soil before the test.

#### 2.3.5. XRD Test and SEM Scanning Test

The XRD and SEM scanning tests carried out using the Institute of Environment and Engineering equipment in cold and arid areas, the Chinese Academy of Sciences. XRD analysis was performed to examine mineral composition and formation of hydration products of the unreinforced and reinforced soil (0% and 0.6%), and 50 g samples were taken from broken samples before and after reinforcement, air dried and passed through a 0.15 mm sieve, ground into powder and made into plane test pieces for observation. SEM was conducted to characterize the micropore structure, morphology, and aggregation state of the soil particles. Before scanning, reinforced and unreinforced soils were sprayed with gold in a vacuum environment to ensure good conductivity in a sputtering machine.

#### 2.3.6. CT Scanning

Computed tomography (CT) technology was used as a sensitive nondestructive 3D imaging technology. The test adopts the nano Voxel-2100 CT device produced by Sanying Precision Instruments Co., Ltd., the device was equipped with a micro-focused closed reflection target X-ray source, a broad vision flat panel detector (427 mm), a mechanical scanning system, digital transmission, and a conversion system. The peak voltage is 225 kV, the maximum power is 320 W, the maximum weight is 25 kg, and the maximum resolution is 0.5 μm. The diameter of the scanned samples is 39.1 mm, and the length is 80 mm.

## 3. Results

### 3.1. Water Stability Test

Collapsible loess is a unique soil due to its pore development, cement dissolution, and soil disintegration properties. The structure is rapidly destroyed when it is soaked by rainfall and snow water, resulting in numerous adverse effects on the stability of the engineering. In order to eliminate this effect, the synthesized stabilizer with different concentrations was used to improve the loess. The disintegration process is shown in Table 3.

**Figure 2:**
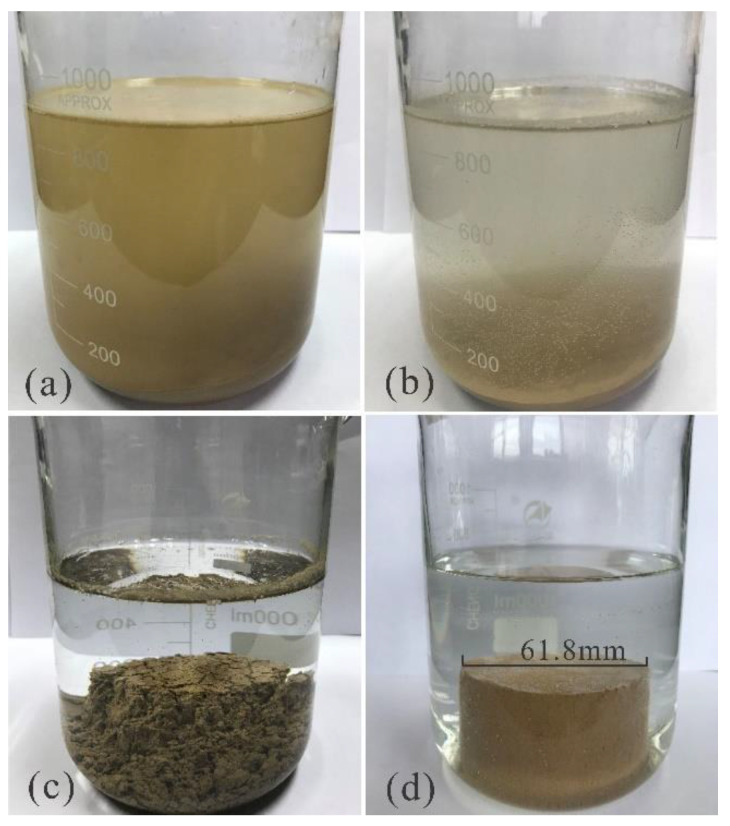
Comparison of the disintegration of the reinforced soil with different stabilizer concentrations: (**a**) 0%; (**b**) 0.2%; (**c**) 0.4%; (**d**) 0.6%.

In the first 0–3 s, the sample absorbed water with a large number of air bubbles, the only difference is that the samples with stabilizer concentrations of 0% and 0.2% dropped numerous soil fragments during the beginning of the test. At 1.5 min, the samples with 0% and 0.2% stabilizer concentrations showed block collapse from different directions, resulting in instantaneous turbidity of the water with in the beaker, while the soil containing 0.4%, 0.6%, and 0.8% stabilizer concentrations was still in the state of water absorption. At 2.5 min, the samples with 0% and 0.2% stabilizer concentrations completely disintegrated, and the samples with a 0.4% stabilizer concentration had a small number of fragments. The samples with 0.6% and 0.8% stabilizer concentration showed no change. At 15 min, the samples with 0% and 0.2% stabilizer concentration collapsed, and the samples with a 0.4% stabilizer concentration presented multiple narrow cracks around the body of the sample. The samples with 0.6% and 0.8% stabilizer concentrations remained unchanged. After 1-h, multiple cracks appeared in the sample containing a 0.4% stabilizer concentration had a small number of blocks fall, and the cracks continued to expand. The samples with stabilizer concentrations of 0.6% and 0.8% remained stable. At 3 h, the soil around the sample with a stabilizer concentration of 0.4% collapsed. However, the samples with stabilizer concentrations of 0.6 and 0.8% remained intact, having only a minimal number of falling fragments and cracks (Figure 2).

**Figure 3:**
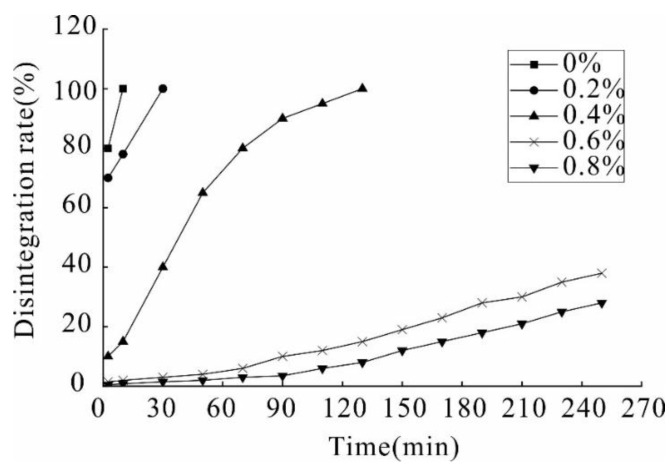
Variations of the disintegration resistance coefficients with different concentration.

The results of the water stability test reveal that during the process of loess strengthening using the stabilizer, the reinforcement effect becomes more apparent as the concentration increases, which is reflected in the following three aspects: First, regardless of stabilizer concentration, the samples will eventually be damaged. However, the time required for specimen failure is significantly different (Figure 3). For example, the samples with 0% and 0.2% stabilizer concentrations were destroyed within 1.5 min. Second, is the difference in water color. The specimens containing low concentrations of stabilizer were damaged in a short time period resulting in turbid water, whereas samples containing high concentrations of stabilizer did not cause water turbidity throughout the test. Third, the shape difference; after the failure of the low concentration samples revealed that the soil is uniform and dispersed at the bottom of the beaker. Finally, the failure of the high concentration samples is distributed around the center of the sample.

### 3.2. Unconfined Compression Test

**Figure 4:**
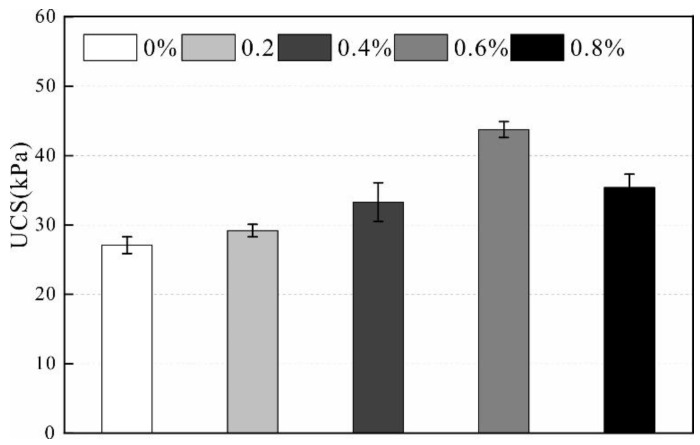
Unconfined compressive strength of wet samples.

Unconfined compressive strength (UCS) is defined as the strength of the specimen against axial deformation without lateral restraint. Therefore, the unconfined compression test is important test for studying the mechanical properties of rocks and soil. In this study, soil strength is improved by adding a stabilizer with different stabilizer concentrations. The relationship between concentration and UCS is obtained and shown in Figure 4.

**Figure 5:**
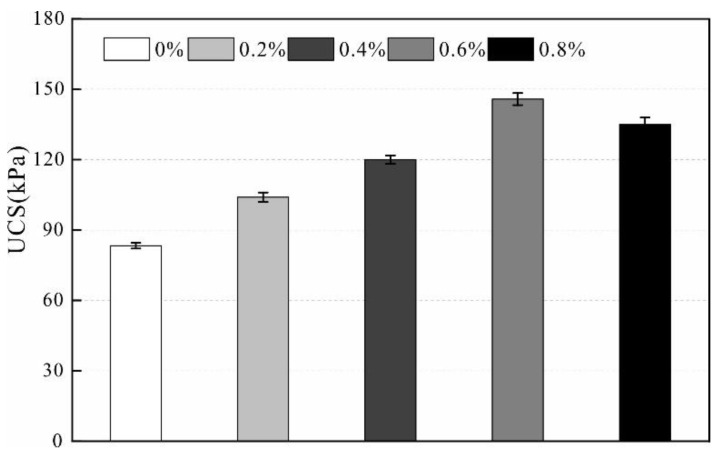
Unconfined compressive strength of dry samples.

As illustrated, the results revealed that the stabilizer effectively improves the strength of the loess. The soil strength gradually increases with increasing stabilizer concentration, especially when the stabilizer concentration changes from 0.4% to 0.6%. The UCS increases rapidly, and the reinforcement effect is the most significant. After the specimen is cured for 24 h under constant humidity, the UCS of the specimen without adding the stabilizer is 27.1 kPa. The solution concentration changes from 0% to 0.2%, the increase in UCS is minimal, and the effect is not apparent. When the solution concentration increases from 0.4% to 0.6%, the UCS is 43.75 kPa. Furthermore, the UCS increased by 61% compared to the sample without the addition of the stabilizer. Therefore, the concentration of 0.4% can be regarded as the critical point of strength mutation. The UCS does not continue to increase with increasing stabilizer concentrations, and the UCS decreases when the solution concentration is 0.8%. The reason for the decrease in strength is that it is difficult for the high concentrations of stabilizer to penetrate uniformly into the sample; thus, the sample is only locally strengthened. After the samples were air dried in indoor conditions, the stabilizer concentration changed from 0% to 0.6%, the UCS increased from 83.3 kPa to 145.8 kPa, and the strength increased by 75% (Figure 5). Therefore, selecting the stabilizer concentration of 0.6% is optimal.

### 3.3. Penetration Test

**Figure 6:**
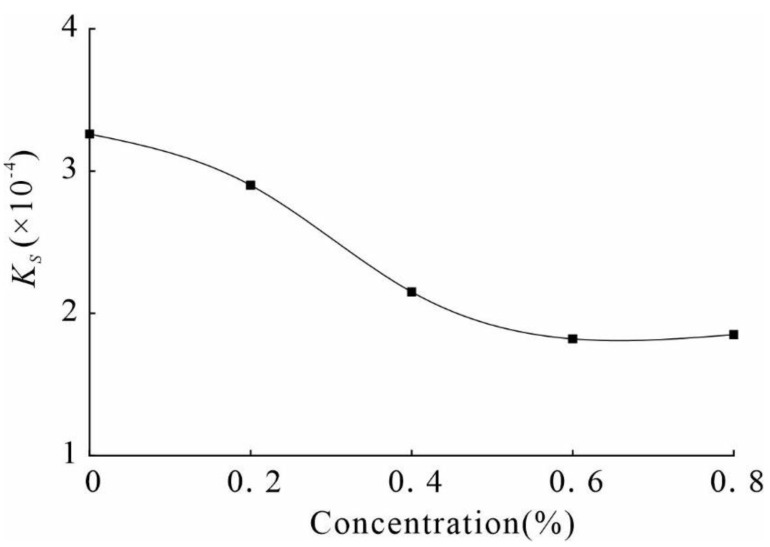
*Ks* of different stabilizer concentrations.

Permeability is a critical engineering parameter of soil and determines the soils strength, deformation, and consolidation properties. Field investigations found that groundwater level rise and water infiltration are significant causes of loess erosion and strength reduction in loess areas. A TST-55 permeameter is used to conduct a variable head permeability test in this study. The *Ks* was measured under different stabilizer concentrations, and the results are shown in Figure 6.

**Figure 7:**
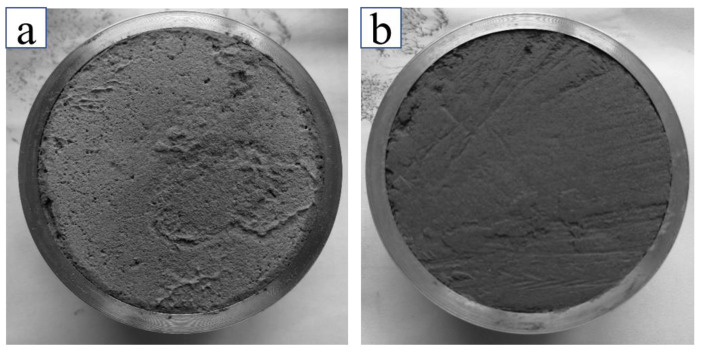
Comparison of penetration samples: (**a**) 0%; (**b**) 0.6%.

It can be observed from Figure 6 that the *Ks* of the reinforced soil decreases significantly, and the *Ks* decreases gradually with an increase in concentration. The *Ks* of the unreinforced soil is 3.26 × 10^−4^, while the *Ks* of the sample reinforced with 0.6% stabilizer is 1.82 × 10^−4^, which is a reduction of 1.79 times. The decrease in the *Ks* indicates that the addition of the stabilizer affects the flow of water in the soil, and is closely related to the pore structure inside the soil as well as the degree of cementation between particles. Additionally, the stabilizer improves the soil’s strength and has minimal impact on its permeability. The comparative analysis of the samples before and after infiltration shows that there are many pores on the surface of the unreinforced sample. However, the soil after treating with the composite stabilizer (0.6%) has a dense surface and no visible pores exist (Figure 7).

### 3.4. Triaxial Test

**Figure 8:**
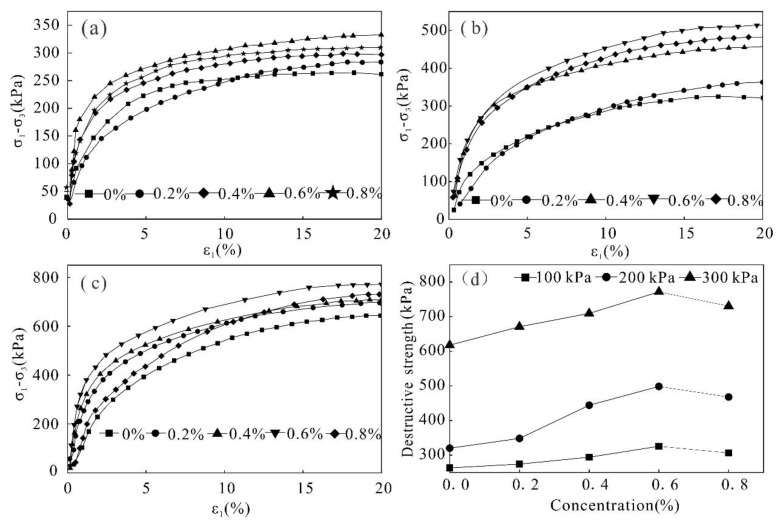
Triaxial compression test curve: (**a**) confining pressure of 100 kPa (**b**) confining pressure of 200 kPa (**c**) confining pressure of 300 kPa and (**d**) destructive strength.

In order to study the shear resistance of the reinforced soil, shear tests were carried out on the soil stabilized with different stabilizer concentrations under three confining pressures of 100 kPa, 200 kPa, and 300 kPa. The stress–strain curves of the soil with different concentrations under the same confining pressures were obtained and shown in Figure 8a–c.

It can be observed from Figure 8 that under the same confining pressures, with an increase in mixed solution concentrations, the corresponding stress–strain curve rises to different extents, and the peak value of the curve gradually increases. When the concentration is greater than 0.6%, the curve suddenly decreases, indicating that the stress will not increase with an increase in concentration, which therefore weakens the reinforcement effect. Furthermore, the soil demonstrates stress hardening, the curve changes from steep to slow, the slope decreases gradually, and there is no apparent peak point. When the concentration is low, the stress–strain curve of soil is close to each other, indicating that the low concentration stabilizer has little effect on the shear resistance of the soil. At the end of the shear test, there is an apparent shear fracture surface on the samples’ surface. However, the samples stabilized by the high concentration stabilizer only show a bulging deformation with no obvious shear fracture on the surface.

The relationship between stabilizer concentration and shear strength had no obvious peak point in the stress–strain curve, and according to the standards of geotechnical test methods [45], the strength corresponding to the strain of 15% is selected as the failure strength of the sample (Figure 8d). Under the confining pressure of 100 kPa, the failure strength of the unreinforced soil is 263.1 kPa, and after reinforcement using a 0.6% stabilizer concentration, the failure strength is 325.6 kPa, thus causing a 23.76% increase in strength. Under a confining pressure of 200 kPa and 300 kPa, the failure strength of the reinforced soil increased by 55.65% and 24.92%, respectively, compared to the unreinforced soil. Furthermore, when the stabilizer concentration is in the range of 0–0.6%, the failure strength of the soil increases with an increase in concentration. Once the concentration exceeds 0.6%, the failure strength of the soil decreases. Therefore, on the premise of ensuring that the solution can easily infiltrate, it is appropriate to select a concentration of 0.6%, where the reinforcement effect tends to be the greatest.

**Figure 9:**
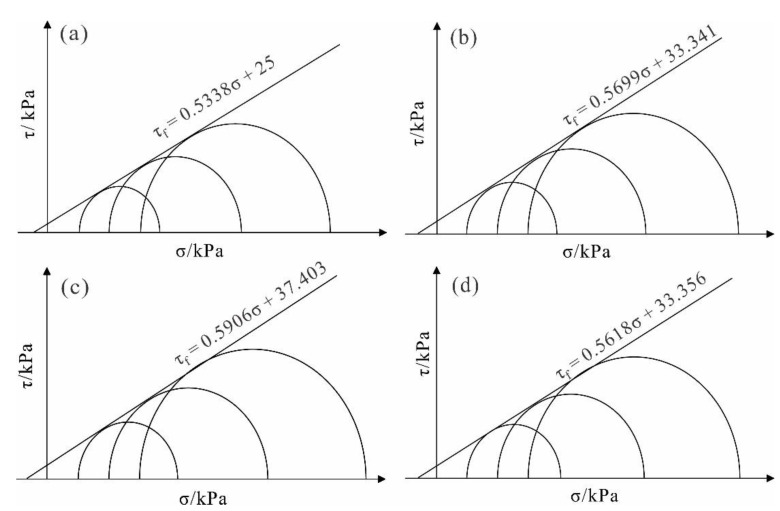
Stress Mohr circles of samples under different stabilizer concentrations: (**a**) 0%; (**b**) 0.4%; (**c**) 0.6%; (**d**) 0.8%.

**Figure 10:**
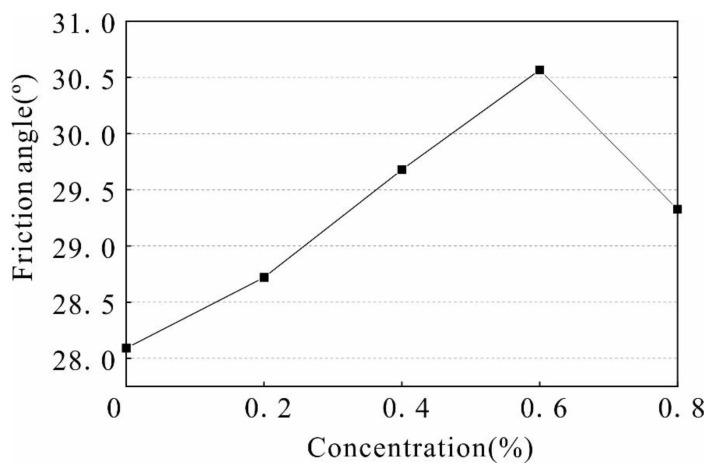
Internal friction angle curve under different stabilizer concentrations.

**Figure 11:**
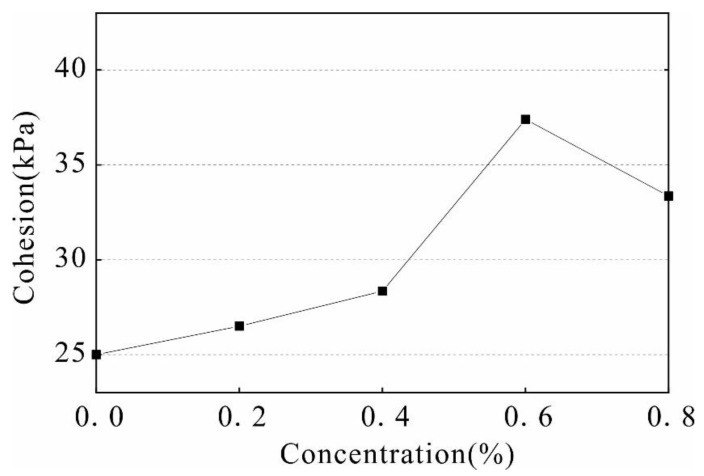
Cohesion curve under different stabilizer concentrations.

The internal friction angle and cohesion are important indexes used to study the shear strength of the soil. Based on the above triaxial test data, the stress Mohr circle and the failure envelope of the solidified soil with different stabilizer concentrations were calculated (Figure 9). The shear strength parameters were obtained as shown in Figure 10 and Figure 11.

As observed from Figure 10 and Figure 11 the internal friction angle and cohesion of the soil increased with an increase in stabilizer concentration. In the range of 0–0.6%, the internal friction angle of the soil increases from 28.09° to 30.57°, and the cohesion increases from 25 kPa to 37.4 kPa, which is an increase of 49.6%. When the stabilizer penetrates into the soil, the stabilizer will agglomerate and cement the dispersed soil particles to form a large number of agglomerates, resulting in an increase in the connection force and cementation between the soil particles. The increase in friction angle and cohesion in varying degrees improves the shear strength of the sample. When the concentration exceeds 0.6%, the friction angle and cohesion decrease due to increased material content of the solution, making it is difficult for the stabilizer to penetrate evenly into the soil. In addition, high concentrations of stabilizer increase the distance between the soil particles, weakening the attraction force between them and causing a rapid decrease in strength.

### 3.5. Erosion Test

In order to evaluate the erosion resistance of the reinforced soil, the natural soil and the reinforced soil (optimal stabilizer concentration of 0.6%) were prepared for the preliminary study. The field survey found that most slopes are severely affected by water, resulting in the formation of a large number of erosion gullies and slope deformation. destroying the artificial slope protection and possible geological disasters. This test was assumed to simulate severe runoff erosion on the slopes caused by heavy rainstorms within the loess area.

**Figure 12:**
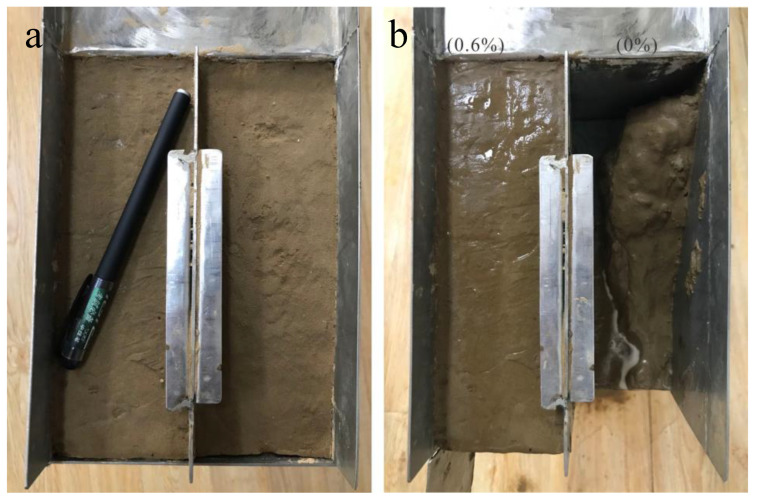
Soil anti-scour test: (**a**) before the test; (**b**) after the test.

As shown in Figure 12, the erosion resistance of reinforced soil was improved due to the incorporation of the composite stabilizer. Initially, when water flows through the slope, part of the water will be absorbed by the unreinforced soil due to loose inter-particle structure and large pore spaces between soil particles; the moisture content of unreinforced soil has a dramatic rise due to the increase in water infiltration. Thus, the capillarity induced inter-particle forces was weakened with the rapid increase in water content, and the untreated soil becomes looser, resulting in the movement and detachment of adjacent particles and the formation of cracks. The formation of cracks has a serious impact on the integrity of the unreinforced soil; the erosion of unreinforced occurred immediately under the action of shear force generated by overland runoff. Therefore, surface runoff and seepage are important factors causing slope erosion. On the contrary, the reinforced soil does not significantly change in the early erosion period. After 20 min, the unreinforced soil has been completely destroyed, and the reinforced soil still has a smooth surface with no visible cracks. At 120 min, the reinforced soil gradually suffered slight damage, and the reinforced soil was not completely destroyed until 4 h.

**Figure 13:**
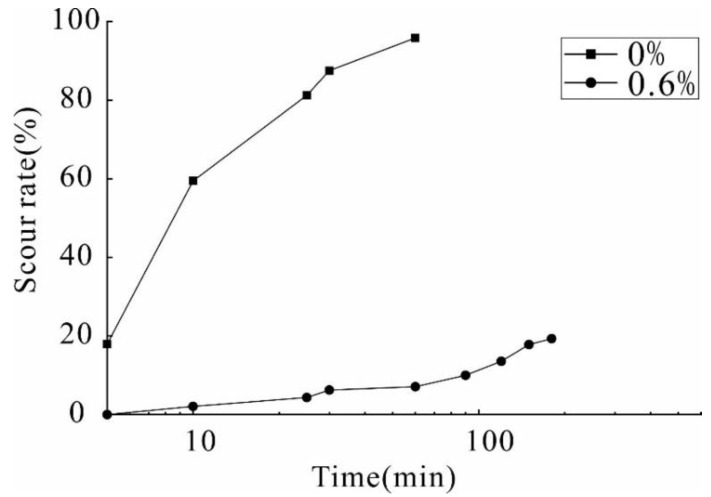
Soil loss curve.

Figure 13 shows the relationship between the cumulative loss ratio and time. The results show that soil loss is different during continuous water erosion. When scouring for 10 min, the loss rate of the unreinforced soil is 59.43%, and that of the reinforced soil is 2.1%, which is a reduction of 28.3 times. After 30 min of scouring, the loss rate of the unreinforced soil reaches 94.5%, and only some soil remains in the test tank, while the loss rate of the treated soil is 6.25%. The loss rate of reinforcement is 15.12 times that of the unreinforced soil, indicating that the stabilizer has a great potential to improve the anti-erosion of the slope. The partially reinforced soil’s loss is due to the soil on the contact surface between the soil and the test device being carried away by the water. In general, the anti-erodibility of the reinforced soil significantly improves, and the reinforcement effect increases.

## 4. Discussion

### 4.1. XRD Chemical Composition Analysis

**Figure 14:**
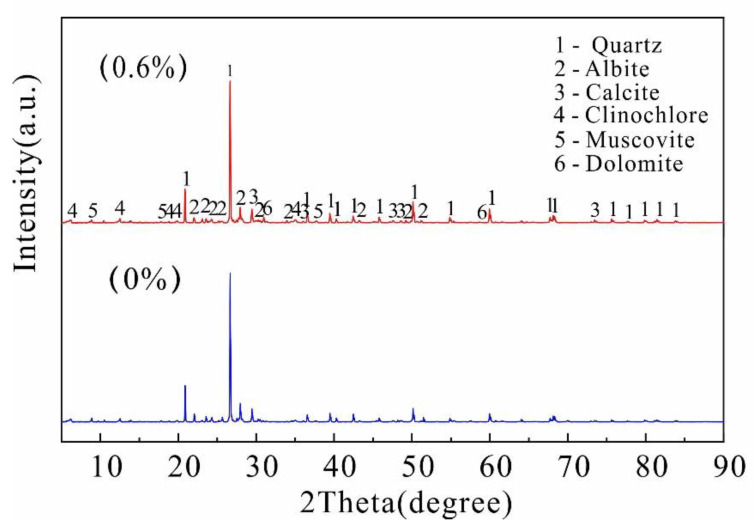
XRD diffraction pattern.

The above test results demonstrate that the stabilizer has a significant reinforcement effect on the soil. The XRD diffraction results were compared before and after reinforcement (Figure 14). The diffraction curve of the treated soil is similar to that of the untreated soil, and no new peak is generated, indicating that the reinforcement process does not generate new substances. The primary clay mineral components are still quartz, calcite, and albite. Although there is no new peak, the peak value of the diffraction curve of the reinforced soil decreases significantly because the solution coats on the surface of the soil particles and inhibits the entry of the rays.

From the analysis of the chemical structure of the reinforcement material, the stabilizer contains water-soluble polymers, and its macromolecular chain contains a large number of hydrophilic functional groups (-COOH, -OH, -CO-NH_2_). The chemical reaction between the polymer and water forms hydrogel, the hydrogel adheres to the surface of the soil particles through van der Waals forces, electrostatic attractions, and hydrogen bonding, therefore forming a stable membrane structure to protect the soil particles from direct contact with the water; the action of the water cannot damage the adhesion between the particles due to the hydrogel, resulting in a significant increase in the water stability of the sample. In addition, these functional groups contain the ligating atoms oxygen and nitrogen, which can undergo complexation reactions with calcium, magnesium, and other metal ions within the loess, resulting in the formation of bridges between the particles. As mentioned above, the chemical changes provide a chemical basis for the stabilization of the loess.

### 4.2. SEM Microscopic Analysis

**Figure 15:**
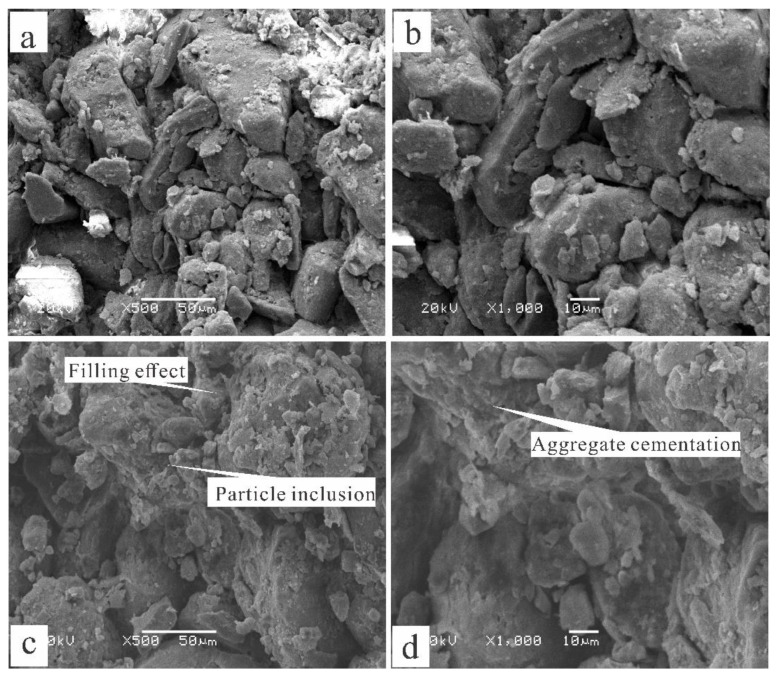
Comparison of SEM images under different stabilizer concentrations: (**a**) 0%, 500 times magnification; (**b**) 0%, 1000 times magnification; (**c**) 0.6%, 500 times magnification; (**d**) 0.6%; 1000 times magnification.

**Figure 16:**
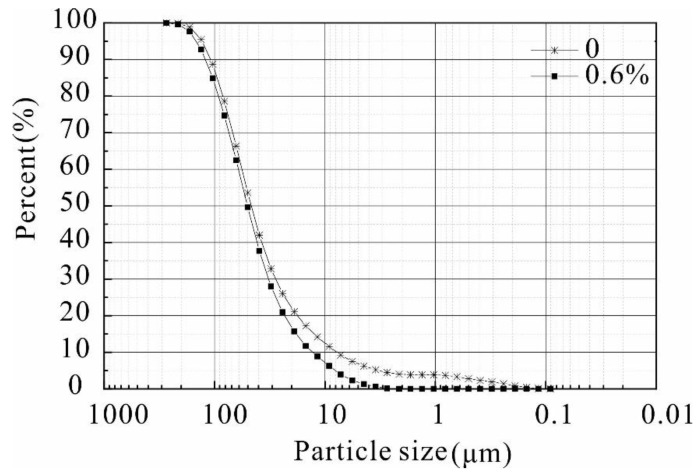
Particle size grading curve of loess with different stabilizer concentrations.

**Figure 17:**
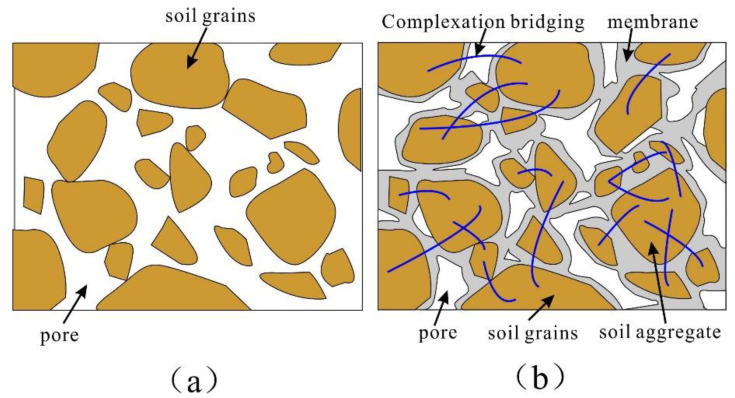
The schematic diagram of effective contact: (**a**) before reinforcement; (**b**) after reinforcement.

The SEM experimental results demonstrate that the particle boundary of the unreinforced soil is clear and distributed in the interior of the soil in a jagged pattern (Figure 15). The stability of the reinforced soil has changed significantly in comparison with the unreinforced soil in regard to the following three aspects. First, when the composite material was added to the loess sample, the polymer attached to the surface of the soil particles and formed an enwrapping, which can fix the soil particles and restrict the movement of the particles due to an increase in the contact area between the particles. This process contributes to the increase in friction strength. Second, the resulting hydrogel has excellent chelation and dispersibility, causing the soil particles to change from a simple point of contact to a surface contact, thus demonstrating agglomeration and cementation that is due to flocculation, so that a single soil particle will form larger particles. Furthermore, the large particles bond with each other to form agglomerates. The formation of larger particles aggregates leads to an increase in soil particle size affecting the particle size grading of the soil. As seen in Figure 16, the particle size grading curve of the soil after reinforcement changes significantly; this process plays a critical role in increasing the cementation strength and stability of the sample. In addition, during the reinforcement process, the complexation reaction has excellent bridging abilities and forms a stable spatial network within the soil, which will greatly improve the cementation and structure of the sample. The schematic diagram of the reinforcement mechanism is shown in Figure 17.

According to Coulomb’s theory, the strength of the soil is composed of friction (*σ tanφ*) and cohesion strength (*c*). Friction strength is affected by particle shape, contact area, particle size gradation and other factors, and cohesion strength depends on various chemical forces and cement content. With the addition of composite materials, various physicochemical effects on friction and cohesion strength greatly improved; therefore, the strength and stability of the soil were significantly improved.

### 4.3. CT Analysis

**Figure 18:**
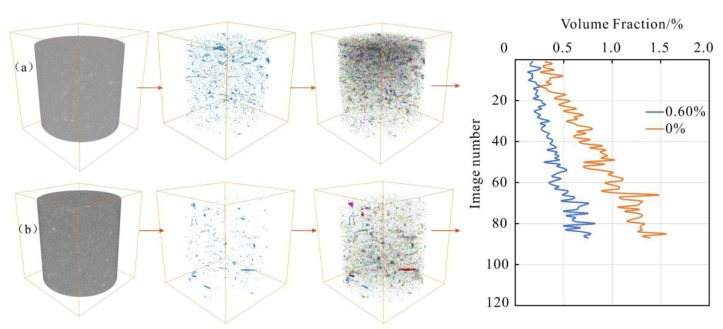
3D pore structure of loess before and after reinforcement: (**a**) 0%; (**b**) 0.6%.

Through the CT image processing and the 3D reconstruction of the pore structure under the same conditions, the 3D pore visual structure model of loess before and after reinforcement is constructed (Figure 18). The number of pores in the soil before reinforcement are numerous and unevenly distributed, while the pores in the soil after reinforcement are significantly reduced and evenly distributed within the soil. As observed in Figure 18, the porosity of the soil after reinforcement decreases along the Z direction, which is in sharp contrast to that before reinforcement. Moreover, the porosity characteristics are consistent with the 3D pore model.

**Figure 19:**
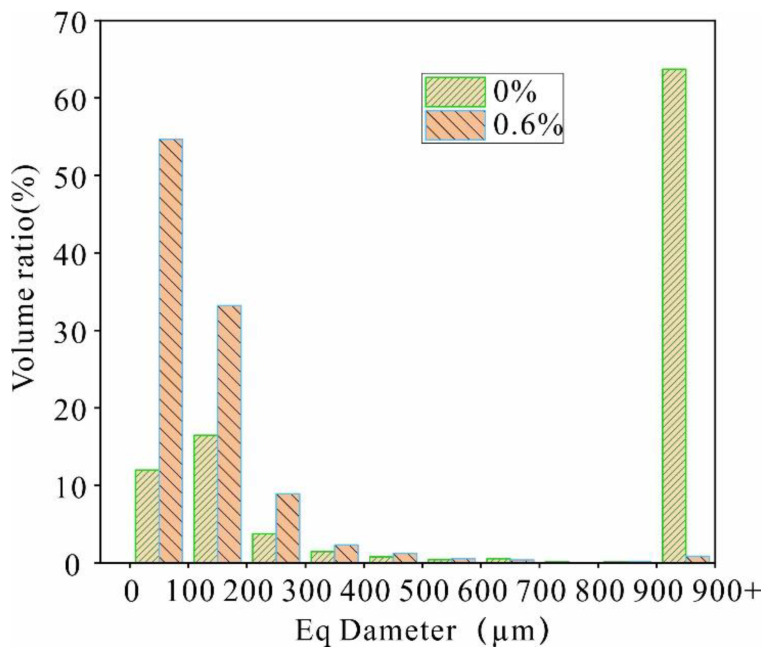
Frequency distribution of the Eq diameter.

**Figure 20:**
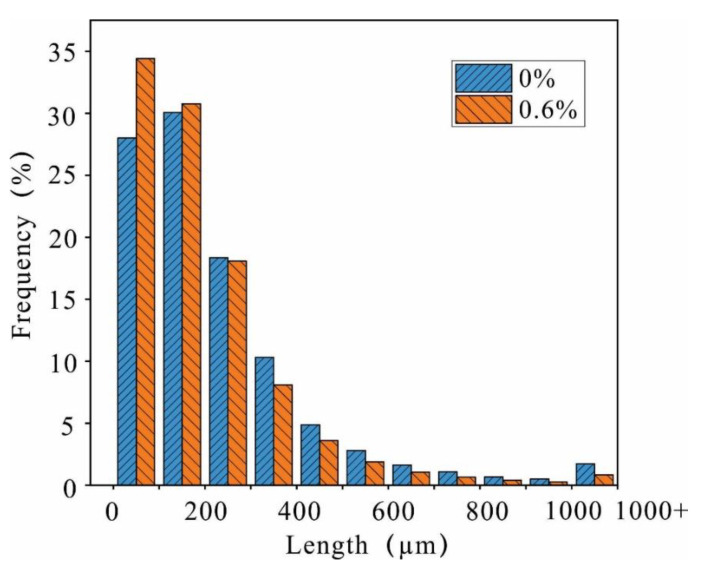
Frequency distribution of the pore length.

To quantitatively describe the pore size and morphology variability induced by the reinforcement process, the percentage distribution of pores with different diameters is presented in Figure 19. After comparison, a large number of pores with a diameter of 900 to 1000 μm are converted to a diameter of 0 to 200 μm, and there is a significant increase in the volume of pores between 0 and 100 μm from 13.3% to 55.6%. Before reinforcement, the proportion of pores with a length of 100–200 μm is the largest; however, after reinforcement, the pores with a length of 0–100 μm are the dominant pore size (Figure 20).

Loess is a multi-phase heterogeneous system with a large number of pores and capillary channels, which facilitates the rapid infiltration of water and the destruction of the soil. Many factors affect the sample’s hydraulic conductivity, such as porosity, pore shape, particle size, and distribution [46]. When the stabilizer was added to the sample, the solution adheres to the particles’ surface through the channels due to its excellent fluidity. The soil particles are first coated, and then the contact mode between the particles changes, eliminating the contact gap between particles and thus, reducing the area of the original pores. Furthermore, filling and cementation are the primary factors leading to the decreased permeability of the reinforced specimen. Due to the excellent film-forming ability of the hydrogel, the inter-particle pores were filled by the membrane structure, reducing the pore volume and pore connectivity, therefore restricting the water flow through limited narrow pores (Table 4). This provides greater resistance to the flow of water and prolongs the permeation path of water, resulting in a significant decrease in the permeability of the sample. In addition, the formation of a denser loess structure is promoted due to the transformation of large pores into relatively smaller pores, leading to the improvement of specimen strength and erosion resistance.

As mentioned above, the composite stabilizer is a new type of environment-friendly soil stabilizer, and can effectively improve the water stability, strength, erosion resistance, and permeability of the loess. When applied in practical engineering, the stabilizer concentration should be 0.6%. Compared to a variety of excellent chemical stabilizers (Table 5) [47], the composite stabilizer also has higher economic benefits, which can provide a theoretical basis for large-scale use in the future.

## 5. Conclusions

In this study, the soil strength, water stability, erosion resistance, permeability, and strength using different stabilizer concentrations were investigated by adding a new and novel stabilizer to the Qingyang Loess within the Gansu Province. The following conclusions were made:

Mixed solutions effectively improve soil erosion resistance and water stability. With an increase in solution concentration, the sample remains intact in water while submerged in water, thus significantly prolonging the disintegration time. After 30 min of continuous water scouring, the slope remained intact after being reinforced with a 0.6% concentration solution. The *Ks* of soil is 1.79 times lower than that of the unreinforced soil.

The improvement effect on strength is most significant when the solution concentration is 0.6%. The unconfined compressive strength of the reinforced soil reaches 145.8 kPa, which is 75% higher than that of the unreinforced soil. Under a 100 kPa, 200 kPa, and 300 kPa confining pressure, the shear strength of soil is 325.6 kPa, 498.4 kPa, and 771.6 kPa, respectively, and increased by 23.76%, 55.65%, and 24.92%, respectively, compared to that of unreinforced soil.

The internal structure of the soil changed due to the reinforcement process. The internal friction angle of the reinforced soil increased from 28.09° to 30.57°, the cohesion changed from 25 kPa to 37.4 kPa, and a significant number of pores with a diameter of 900–1000 μm reduced to a diameter of 0–200 μm. Some of the pores with a length greater than 600 μm were reduced into pores with a size less than 200 μm. The dispersed particles in the soil form aggregates; therefore, changes in the microstructure led to a change in water stability and strength.

## Figures and Tables

**Table 1:** Physical property parameters of the loess.

Parameters	Values
Natural moisture content (%)	4.52
Maximum dry density(g·cm^−3^)	1.85
Specific gravity (g·cm^−3^)	2.71
Plastic limit (%)	17.7
Liquid limit (%)	38.63
Optimum moisture content (%)	21.17

**Table 2:** Properties of the reinforcement materials.

Component	Solid Form	Dissolution Time (h)	Solution Volatility (%)	Molecular Weight
A	White granular powder	<0.01	<1	748.80
B	White powder	<0.5	<1	902.93
C	White columnar crystal	<0.3	<1	258.07

**Table 3:** Sample disintegration process.

Material Concentration/﹪	Disintegration Process					
	3 s	1.5 min	2.5 min	15 min	1 h	3 h
0	○▲	★▼	□	━	━	━
0.2	○▲	★▼	□	━	━	━
0.4	○	○▲	▲	●	□	━
0.6	○	○	▲	●	●▼	▼
0.8	○	○	━	━	━	━

Note: “○” Indicates water absorption and bubbling; “★” Water turbidity; “●” Crack growth; “▲” Chip dropping; “▼” Massive collapse; “□” Complete disintegration; “━” unchanged.

**Table 4:** Comparison of the 3D structural parameters.

Concentration (%)	Overall Volume Fraction (%)	Specific Surface Area (μm^2^/μm^3^)	Connected Porosity (%)
0%	8.92	0.06	-
0.6%	3.93	0.05	-

**Table 5:** Costs of various soil stabilizer.

Type	Unit Price (USD/kg)	Cost (USD/m^3^)
Polyacrylamide	3.0	225
Carboxymethyl cellulose	1.1	33
Xanthan gum	2.9	87
Hydroxypropyl methylcellulose	2.4	36
Synthetic resins	2.9	87
Polymer (OPS)	0.7	32.9
Composite stabilizer	2	26.2

## Data Availability

Data presented in this study are available on request from the first author.
